# Phenotypic and molecular insights into *CASK*-related disorders in males

**DOI:** 10.1186/s13023-015-0256-3

**Published:** 2015-04-12

**Authors:** Ute Moog, Tatjana Bierhals, Kristina Brand, Jan Bautsch, Saskia Biskup, Thomas Brune, Jonas Denecke, Christine E de Die-Smulders, Christina Evers, Maja Hempel, Marco Henneke, Helger Yntema, Björn Menten, Joachim Pietz, Rolph Pfundt, Jörg Schmidtke, Doris Steinemann, Constance T Stumpel, Lionel Van Maldergem, Kerstin Kutsche

**Affiliations:** Institute of Human Genetics, Heidelberg University, Heidelberg, Germany; Institute of Human Genetics, University Medical Center Hamburg-Eppendorf, Martinistraße 52, 20246 Hamburg, Germany; CeGaT GmbH, Tübingen, Germany; Universitätskinderklinik, Universitätsklinikum Magdeburg, Magdeburg, Germany; Klinik und Poliklinik für Kinder- und Jugendmedizin, Neuropädiatrie, Universitätsklinikum Hamburg-Eppendorf, Hamburg, Germany; Department of Clinical Genetics and School for Oncology & Developmental Biology (GROW), Maastricht UMC+, Maastricht, The Netherlands; Klinik für Kinder- und Jugendmedizin, Universitätsmedizin Göttingen, Göttingen, Germany; Department of Human Genetics, Radboud Institute for Molecular Life Sciences, Radboud University Medical Center, Nijmegen, The Netherlands; Center for Medical Genetics, Ghent University, Ghent, Belgium; Section of Neuropediatrics, Center for Child and Adolescent Medicine, Heidelberg, Germany; Institut für Humangenetik, Medizinische Hochschule Hannover, Hannover, Germany; Institut für Zell- und Molekularpathologie, Medizinische Hochschule Hannover, Hannover, Germany; Centre de Génétique Humaine, Université de Franche-Comté, Besançon, France

**Keywords:** CASK, Microcephaly, Nystagmus, Pontocerebellar hypoplasia, X-linked intellectual disability

## Abstract

**Background:**

Heterozygous loss-of-function mutations in the X-linked *CASK* gene cause progressive microcephaly with pontine and cerebellar hypoplasia (MICPCH) and severe intellectual disability (ID) in females. Different *CASK* mutations have also been reported in males. The associated phenotypes range from nonsyndromic ID to Ohtahara syndrome with cerebellar hypoplasia. However, the phenotypic spectrum in males has not been systematically evaluated to date.

**Methods:**

We identified a *CASK* alteration in 8 novel unrelated male patients by targeted Sanger sequencing, copy number analysis (MLPA and/or FISH) and array CGH. *CASK* transcripts were investigated by RT-PCR followed by sequencing. Immunoblotting was used to detect CASK protein in patient-derived cells. The clinical phenotype and natural history of the 8 patients and 28 *CASK*-mutation positive males reported previously were reviewed and correlated with available molecular data.

**Results:**

*CASK* alterations include one nonsense mutation, one 5-bp deletion, one mutation of the start codon, and five partial gene deletions and duplications; seven were *de novo*, including three somatic mosaicisms, and one was familial. In three subjects, specific mRNA junction fragments indicated in tandem duplication of *CASK* exons disrupting the integrity of the gene. The 5-bp deletion resulted in multiple aberrant *CASK* mRNAs. In fibroblasts from patients with a *CASK* loss-of-function mutation, no CASK protein could be detected. Individuals who are mosaic for a severe *CASK* mutation or carry a hypomorphic mutation still showed detectable amount of protein.

**Conclusions:**

Based on eight novel patients and all *CASK*-mutation positive males reported previously three phenotypic groups can be distinguished that represent a clinical continuum: (i) MICPCH with severe epileptic encephalopathy caused by hemizygous loss-of-function mutations, (ii) MICPCH associated with inactivating alterations in the mosaic state or a partly penetrant mutation, and (iii) syndromic/nonsyndromic mild to severe ID with or without nystagmus caused by *CASK* missense and splice mutations that leave the CASK protein intact but likely alter its function or reduce the amount of normal protein. Our findings facilitate focused testing of the *CASK* gene and interpreting sequence variants identified by next-generation sequencing in cases with a phenotype resembling either of the three groups.

**Electronic supplementary material:**

The online version of this article (doi:10.1186/s13023-015-0256-3) contains supplementary material, which is available to authorized users.

## Background

In 2008, we found the X-linked *CASK* gene (MIM 300172) to be mutated in female patients with a severe neurodevelopmental disorder and distinct brain anomalies which comprised progressive microcephaly, pontocerebellar hypoplasia and severe developmental delay (DD), named microcephaly with pontine and cerebellar hypoplasia (MICPCH, MIM 300749) [1]. Subsequently, the MICPCH phenotype has been well delineated by several groups [2-5]. Affected females show primary or rapidly postnatally evolving microcephaly, and typically (moderate to) severe DD/intellectual disability (ID) with no or very limited language. Facultative features are axial hypotonia and/or peripheral hypertonia, movement and behavioral disorders, and seizures [6]. These patients inconsistently show short stature, various eye anomalies, sensorineural hearing loss, and possibly a recognizable facial phenotype, whereas congenital malformations are rarely associated [2,4]. MRI findings are characteristic and allow differentiation of other microcephalic disorders, in particular of the different types of pontocerebellar hypoplasia (PCH). In MICPCH, cerebellar hypoplasia is diffuse affecting the hemispheres and vermis equally, and pontine hypoplasia can show relative sparing of the pontine bulging. Both pontine and cerebellar hypoplasia can be mild to severe without correlation to the severity of clinical manifestations [4,5]. The corpus callosum (CC) is of normal or low-normal size, exhibits a low cerebrum/CC ratio [7], and supratentorial anomalies are subtle and non-specific possibly showing a rarefication of gyri in the frontal region. Females with MICPCH carry heterozygous loss-of-function mutations in *CASK* including nonsense, frameshift, and splice site mutations as well as partial or complete deletions/duplications of *CASK* [1-5].

So far, only six males with likely loss-of-function alterations of *CASK* have been reported [2,5,8,9], all presenting with a severe neurological phenotype except for one who had a mosaic mutation. In addition, familial cases of male patients with missense and splice mutations in *CASK* have also been described. They exhibit mild to severe ID with or without nystagmus or were diagnosed with the so-called FG-syndrome [10-12]. It has been hypothesized that *CASK* loss-of-function mutations are associated with reduced male viability or *in utero* lethality [1], while hypomorphic mutations are associated with a different phenotypic spectrum [10]. However, the phenotype in males associated with different *CASK* mutation types has not been systematically evaluated.

We here report on eight unrelated male individuals, seven sporadic and one familial case, with different *CASK* alterations (nonsense and splice mutations, mutation of the start codon, partial gene duplications and deletions, also in mosaic state), and evaluate the clinical, genetic and molecular data of these novel subjects and the male individuals published previously. We also review the current knowledge on pathogenic mechanisms and genotype-phenotype correlation in individuals with a *CASK* mutation.

## Methods

### Patients

Clinical and molecular findings in patients 1–8 as well as in previously published male patients with a *CASK* alteration are summarized in Table 1. The clinical data and samples were obtained with informed consent of the patients’ parents/guardians, including consent to use the photographs in this report, according to the Declaration of Helsinki and the national legal regulations (e.g. German Genetic Diagnosis Act [GenDG]).

**Table 1:** **Male individuals with** ***CASK*** **mutations**

**Patient**	***CASK*** **mutation**	**Birth OFC/W/L (SD)**	**Age**	**OFC** ^**a**^ **(SD)**	**Height** ^**a**^ **(SD)**	**Weight** ^**a**^ **(SD)**	**DD/ID**	**Tonus**	**Seizures EEG**	**Other neurologic anomalies**	**Eye findings**	**Sensorineural hearing loss**	**Other anomalies**	**Face**	**MRI**	**Overall phenotype**
**Pat 1**	c.704_708del p.K236Efs*10 ex 7 dn	w 37 +3 -4.2/-3.2/?	† at 7 m	−5.9 (4.5 m)	−4.8 (6 m)	−1 (6 m)	profound, no development	severe hypotonia	intractable seizures	apnoeas, inability to swallow	optic atrophy	+	ASD, bil. clubfeet	dolichocephaly, puffy eyelids, broad nasal bridge, bulbous tip of nose, severe retromicrognathia, ear dysplasia, fleshy ear lobes	severe hypo CBL + pons + medulla, simplified gyri, cortical atrophy	**MICPCH Severe epilepsy**
**Pat 2**	Dup ex 10–16 dn	w 37 +3 -2.5/-1.9/-1.5	10 m † at 21 m	progressive microcephaly	−2.4	−2.8	profound	severe hypotonia	probably Ohtahara s., burst suppression		macropapilla, optic atrophy?	+?	long convex fingernails, overriding 2nd toes, linear blisters right leg, needed PEG	retrognathia, fleshy uplifted ear lobules	significant hypo CBL + pons	**MICPCH Severe epilepsy**
**Pat 3**	c.1A > G ex 1 dn	−1.3/-0.3/?	5 y	−5 (3 y)	−3	−2	profound, no development	severe hypotonia	intractable seizures		opticus hypoplasia	–	AVSD, tapering fingers, edema of the dorsum of hands and feet, needed PEG	long eyelashes, short nose, large ears with fleshy uplifted ear lobules	small brain, hypo CBL + pons	**MICPCH Severe epilepsy**
				−3.7 (5 y)												
**Pat 4**	c.79C > T p.R27* ex 2 dn	w 33 +2 -2.57/-1.14/-1.73	15 m	−9.0	−3.0	−1.67	profound	severe hypotonia	intractable seizures, burst suppression	apnoea-bradycardy-syndrome, inability to swallow	optic atrophy?		VSD, short limbs, contractures of fingers	plagiocephaly, metopic ridge	severe hypo CBL + pons, progressive cortical atrophy, progressive hypomyelination	**MICPCH Severe epilepsy**
**Pat 5**	Dup ex 4–20 mos	w 36 +1 -1.8/0/0.3	7 m	−7.8 (9 m)	−2.1 (9 m)	−0.6 (9 m)	severe	hypertonia	spasms and myoclonic seizures, no hypsarrhythmia	–	hyperopia	?	micropenis, cryptorchidism	sparse hair, broad nasal bridge, epicanthal folds, long philtrum, retromicrognathia, fleshy uplifted ear lobules	small brain, hypo CBL + pons	**MICPCH epilepsy**
**Pat 6**	Del ex 1 mos	−2.9/-1.1/-1.7	16 m	−6 (11 m)	−2 (11 m)	−1.6 (11 m)	severe	hypertonia of limbs	–	–	hyperopia, strabism	–	cryptorchidism	broad nasal bridge, epicanthal folds, long philtrum, prominent premaxilla, mild retrognathia, simple/thin auricle	hypo CBL + pons	**MICPCH**
**Pat 7**	Del ex 3–9 mos	w 37–0.93/-0.5/-0.87	29 m	−3.56 (29 m) -2.55 (5;3 y)	−3.5 (29 m) -2.42 (5;3 y)	−1.97 (29 m) -1.45 (5;3 y)	severe	tonus regulation disorder	–	mild ataxia	–	–	–	prominent nasal bridge, thin upper lip, pointing chin	mild hypo CBL + pons mildly simplified gyri	**MICPCH**
**Pat 8**	Dup ex 1–5 mat	−2 /0/-1.4	20 m	−5	−2	−2	Mild to moderate DD		–	–			FTT	long flat philtrum	mildly smaller frontal lobes, small CC (frontal), CBL and otherwise normal	**MIC + DD**
Pat 16^b^	c.1061T > C p.L354P^c^ ex 12		5 y				profound, no development		West s., intractable seizures					large eyes, large ears, broad nasal bridge, broad nasal tip, epicanthal folds^d^	MICPCH	**MICPCH Severe epilepsy**
Pat 1^e^	Del ex 2 mat^f^	−1.2/-2/-1.4	4 y	−2.7 (1.4 y)			profound		Ohtahara s.				Long slender fingers, micropenis, needed tracheostomy + PEG	micrognathia, short neck	severe hypo CBL, hypo pons^d^	**MICPCH Severe epilepsy**
Pat 2^e^	c.1A > G ex 1 dn	−2.7/-3.3/-2.8	4 y				severe	hypertonia of limbs	Ohtahara s.		PHPV	–	short upper arms, overlapping fingers, clinodactyly	micrognathia, high arched palate	severe CBL hypo, hypo pons^d^	**MICPCH Severe epilepsy**
Case report^g^	c.227_228del p.E76Vfs*6 ex 3 dn	0/0.1/1.4	8 m	−5.4			severe	hypotonia	early myoclonic encephalopathy (EME)	dystonia, chorea	optic atrophy		CP, tetralogy of Fallot, AMC, hydronephrosis, VUR, needed tracheostomy	micrognathia	severe hypo CBL + pons	**MICPCH Severe epilepsy**
Pat 13^h^	c.278 + 1G > A in 3 dn	−2/0/-0.5	16 m	−6	−2		profound	profound hypotonia	intractable seizures, spasms + tonic seizures, suppression-burst	spastic tetraparesis, dystonia	optic atrophy		long slender fingers with contractures, needed PEG	retrognathia, high arched palate, low-set ears with prominent lobules, down-slanted palpebral fissures, broad nasal bridge	very severe hypo CBL, hypo pons	**MICPCH Severe epilepsy**
Pat 12^h^	c.316C > T p.R106* ex 4 mos	−3/-1.5/-2	15 y	−3.5	−3.5		severe		–	dystonia, dyskinesia	–	–		well-arched eyebrows, broad nasal bridge, hypertelorism?, anteverted nares, full lips^d^	mild hypo CBL	**MICPCH**
Pat 5^i^	c.915G > A p.(=) ex 9 dn		† at 2 w	microcephaly					?						severe hypo CBL + pons, thin and unmyelinated CC	**MICPCH**
Pat II 4^j^	Fam V c.2183A > G p.Y728C ex 23 (2 ♂)		14 y	−4.4^k^	−2.9	thin	severe				N, strabism, optic atrophy			synophris, high nasal bridge, upslanted palpebral fissures, short columella^d^	hypo CBL, pachygyria	**MICPCH + N**
Pat II 2^j^			19 y	−2.4^k^	−1.7	thin	moderate				N, astigmatism			similar to II 4	ND	**MIC + Moderate ID + N**
Pat IV 1^j,l^	Fam 74 c.2129A > G p.D710G ex 22 (4 ♂)		42 y	normal	normal		mild			hand tremor	N, strabism			no dysmorphism	ND	**Mild ID + N**
Pat III 3^j,l^				98th cen	normal	obese	mild			tremor, unsteady gait	N, strabism				ND	**Mild ID + N**
Pat III 12^j,l^				98th cen	normal	obese	mild				N				normal	**Mild ID + N**
Pat III 6^j,l^			59 y	normal	normal		mild							no dysmorphism	ND	**Mild ID**
Pat IV 1^j,l^	Fam 16 c.802T > C p.Y268H ex 8 (4 ♂, 1 without clinical description)			normal	normal		severe/profound		+	toe walker	no N		autistic	no dysmorphism	ND	**Severe ID**
Pat III 4^j,l^							severe		+		no N				ND	**Severe ID**
Pat II 3^j,l^							severe			toe walker	no N		obsessive behaviour		ND	**Severe ID**
three Pat^j,l^	Fam 123 c.2756T > C p.W919R ex 27 (3 ♂)			normal	tall		mild		+, in one of three patients at age 17 y		N			no dysmorphism	ND	**All 3: Mild ID + N**
Pat III 1^j,l^	Fam 245 (K8919) c.1186C > T p.P396S ex 13 (4 ♂, 2 without clinical description)		58 y	0	short stature, 152 cm		profound			tremor, unsteady gait				flat mid face, open mouth, eversion of lower lip	ND	**Severe IDS**
Pat III 4^j,l^			52 y	0	short stature, 147 cm		severe				strabism			flat nasal bridge, anteverted nares, wide mouth, broad palate, broad grooved tongue, short broad neck	ND	**Severe IDS**
Pat III 3^j^	Fam 683 c.2521-2 A > T in 25 (4 ♂)		46 y	+1.6	−2.2		mild		+, absences	–	N, high myopia			no dysmorphism	ND	**Mild ID + N**
Pat II 3^j^							mild		+		N, strabism, myopia, astigmatism				ND	**Mild ID + N**
Pat II 4^j^							mild		–		N, high myopia, optic atrophy, retinal pigment anomalies				ND	**Mild ID + N**
Pat II 5^j^							mild		+		N				ND	**Mild ID + N**
Pat III 26^m^	c.83G > T p.R28L ex 2 (3 ♂)		2 y	relative macrocephaly	50-75th cen	25-50th cen	marked	hypotonia	EEG mildly abnormal		–	+	hyperactivity, aggressive behaviour, constipation	prominent forehead, frontal upsweep, hypertelorism, depressed nasal bridge, long philtrum, micrognathia^d^	normal (on CT)	**IDS**
Pat II 11^m^			34 y	relative macrocephaly			profound	hypotonia in infancy	+				aggressive behaviour, constipation	prominent forehead, hypertelorism, broad long philtrum^d^	ND	**IDS**
Pat II 17^m^			16 y		short stature		+, unspecified	hypotonia in infancy				+	hyperactivity, constipation, cryptorchidism	prominent forehead, frontal upsweep, long philtrum, epicanthal folds^d^	ND	**IDS**

**Legends:** The eight first listed patients (Pat 1-8 in bold) are described in detail in the manuscript, patients listed below have been reported previously. +, present; −, absent; †, died; AMC, arthrogryposis multiplex congenita; ASD, atrial septal defect; AVSD, atrioventricular septal defect; bil., bilateral; CBL, cerebellum; CC, corpus callosum; cen, centile; CP, cleft palate; CT, computed tomography; DD, developmental delay; Del, deletion; dn, *de novo*; Dup, duplication; EEG, electroencephalogram; ex: exon; F, female; Fam, family; FTT, failure to thrive; hypo, hypoplasia; ID, intellectual disability; IDS, syndromic intellectual disability; in, intron; L, length; m, months; mat, maternally inherited; MIC, microcephaly; MICPCH, microcephaly with pontine and cerebellar hypoplasia; mos, somatic mosaicism; MRI, magnetic resonance imaging; N: nystagmus; ND, not documented; OD, right eye; OS, left eye; Pat, patient; PEG, percutane g-tube; PHPV, persistent hyperplastic primary vitreous; s., syndrome; SD, standard deviation; VSD, ventricular septal defect; VUR, vesicoureteral reflux; W, weight; w, week of gestation; y, year(s).

^a^at last follow up (if not specified otherwise).

^b^Takanashi *et al*., 2012 [5].

^c^in original publication wrongly reported as p.L348P.

^d^based on figures/photographs shown in publication.

^e^Saitsu *et al*., 2012 [9].

^f^the mother is a somatic mosaic for the *CASK* mutation.

^g^Jinnou *et al*., 2012 [18] and Nakamura *et al*., 2014 [8].

^h^Burglen et al., 2012 [2].

^i^Najm *et al.*, 2008 [1].

^j^Hackett *et al*., 2010 [10].

^k^in Table 1 of the original publication wrongly listed as normocephaly (absolute values were given in the text).

^l^Tarpey *et al*., 2009 [12].

^m^Piluso *et al*., 2003 [19] and 2009 [11].

**Figure 1:**
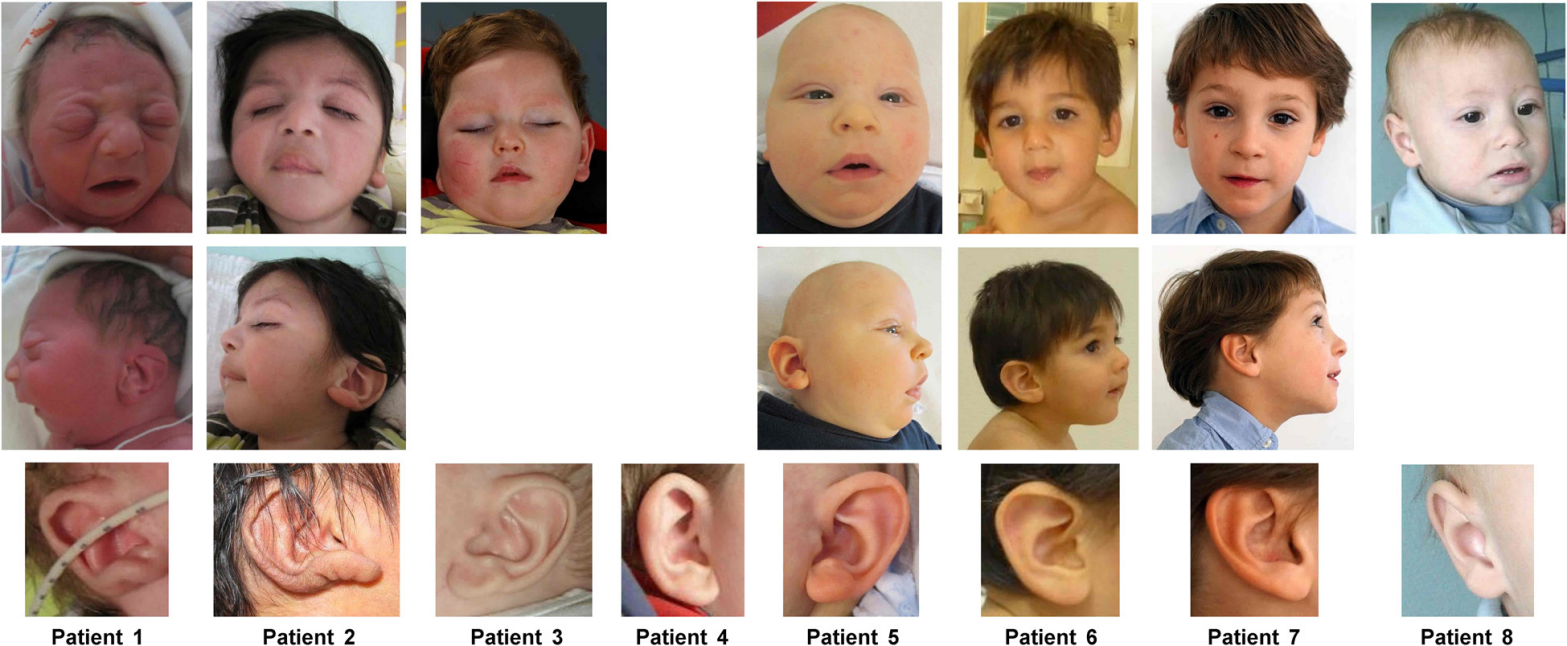
Photographs of patients 1–8. Facial features of patients 1–8 are shown at the age of 1 day (patient 1), 9 months (patient 2, ear at 3 days), 19 months (patient 3), 7 months (patient 5), 16 months (patient 6), 5 years (patient 7), 14 months (patient 8), ears are depicted in the bottom row. Apart from microcephaly, patients 5 and 6 show epicanthal folds and a long philtrum, patient 8 a flat smooth philtrum, patients 1, 5 and 6 a broad and patient 7 a prominent nasal bridge, patient 1 a bulbous tip of nose, patients 1 and 5 retromicrognathia, and patients 1, 2, 3 and 5 fleshy, uplifted ear lobules. There seems to be no recognizable facial phenotype.

**Figure 2:**
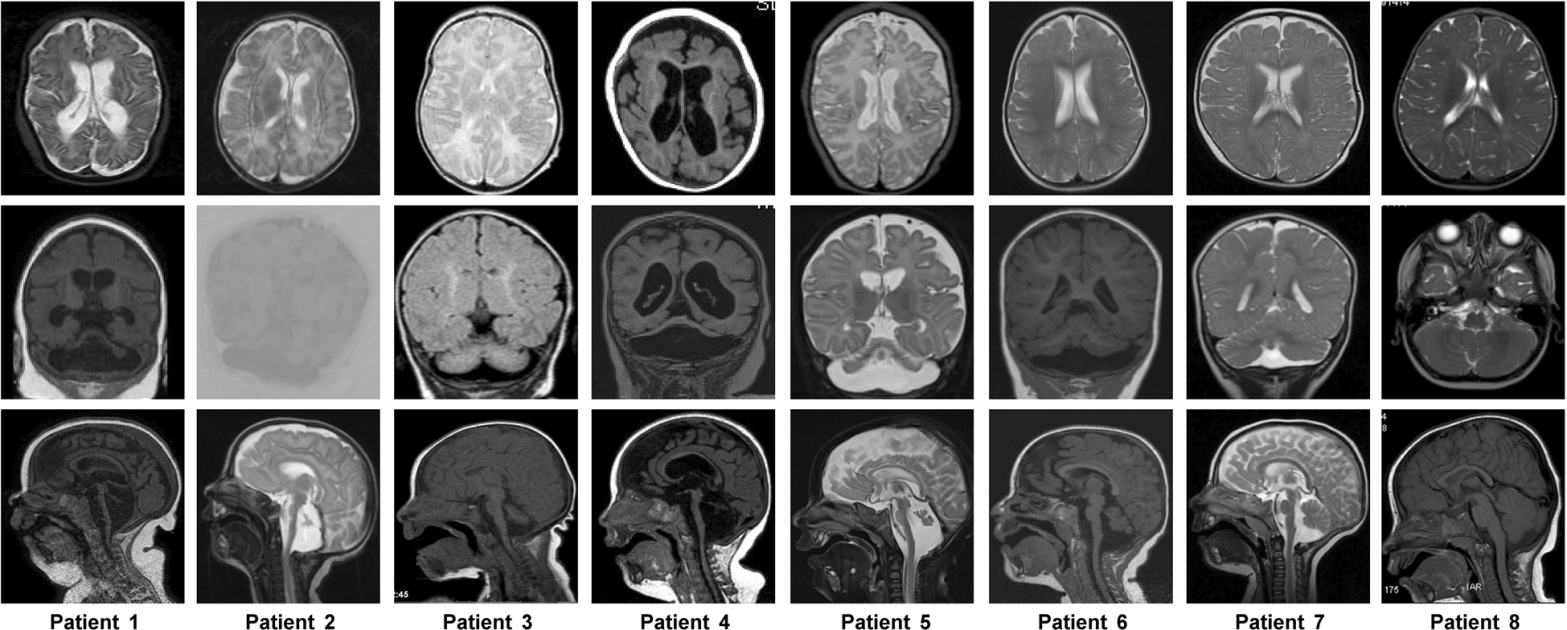
Selected axial, coronal and sagittal MR images from eight male individuals with a *CASK* alteration. The coronal images (second row) show hypoplastic, flattened cerebellar hemispheres with proportionally reduced size of the vermis in patients 1–7. The sagittal images in the third row show intact corpus callosum in all cases, low forehead indicative for microcephaly and pontine hypoplasia in patients 1–7. Pontocerebellar hypoplasia is severe in patients 1–5, moderate in patient 6, and mild in patient 7. Cerebellum and pons of patient 8 are normal. A mildly reduced number and complexity of the frontal gyri are seen in patients 1 and 7, and cortical atrophy in patients 1 and 4 (axial images in first row). MR imaging was performed at the age of 6 months (patient 1), 5 months (patient 2), 5 years (patient 3), 2 months (patient 4), 4 months (patient 5), 11 months (patient 6), 10 months (patient 7), and 16 months (patient 8).

**Patient 1** was born in week 37 as the 1st child of Dutch parents. He had primary microcephaly (−4.2 SD), bilateral clubfeet which had already been noticed during pregnancy, and craniofacial dysmorphism consisting of dolichocephaly, puffy eyelids, broad nasal bridge, bulbous tip of the nose, severe retromicrognathia, and ear dysplasia with fleshy uplifted ear lobules (Figure 1). Apgar scores were 3, 5 and 10 at 1, 5 and 10 minutes, respectively. He had a severe neurologic disorder with hypotonia, abnormal movements, inability to swallow and intractable, probably myoclonic seizures. The EEG was severely abnormal with multifocal abnormalities and slow discontinued and flat episodes. On cranial MRI, severe hypoplasia of medulla, pons and cerebellum, in particular of cerebellar hemispheres, progressive cortical atrophy, simplified gyri and hypomyelination were seen (Figure 2). Ophthalmologic examination revealed optic hypoplasia, BERA severe sensorineural hearing loss, and cardiologic examination a large ASD I, a small ASD II, and atrioventricular valve insufficiency 2/4. He suffered from severe apneas and chronic hypoventilation for which he received CPAP and caffeine. At age 7 months, he developed an airway infection and died due to respiratory insufficiency despite antibiotic treatment and respiratory support.

**Patient 2** was the 7th child of an unrelated Pakistani couple. The pregnancy was complicated by polyhydramnios, gestational diabetes controlled by a diet and IUGR. He was born in week 37 with mild microcephaly (−2.5 SD), small for gestational age, and required cardiopulmonary reanimation because of cyanosis and lack of spontaneous breathing. He was severely hypotonic and developed myoclonic seizures from his 1st month of life, later tonic and infantile seizures which were difficult to treat. His EEG showed a burst-suppression-pattern and a probable diagnosis of Ohtahara syndrome was established. Because of swallowing difficulties he was fed by a PEG tube but continued to be dystrophic. He showed nearly no development, and was last seen at the age of 10 months. Dysmorphic features consisted of retrognathia, fleshy uplifted ear lobules, long convex fingernails, and overriding 2nd toes (Figure 1). He died at the age of 21 months, probably from pneumonia. MRI brain showed significant hypoplasia of the pons and all parts of the cerebellum. No supratentorial anomalies were seen except a retardation of myelination (Figure 2).

**Patient 3** was born as the 1st child of Dutch parents. The pregnancy was established with IVF/ICSI with frozen sperm which was obtained before the start of chemotherapy because of a hematologic malignancy in the father, and proceeded uneventfully. An ASD with bicuspid aortic valve was diagnosed shortly after birth. OFC at birth was within the normal range but rapidly decreased to −2.5 SD at the age of 3 months and −5 SD at age 3 years. He showed minor dysmorphic facial features with fleshy earlobes (Figure 1). From the age of 2 weeks on epileptic attacks were noted. Despite antiepileptic treatment he persisted having severe intractable seizures. He was severely hypotonic and had feeding problems, needing a PEG tube at age 2 years. He showed virtually no motor or cognitive development. He was able to recognize his parents in periods when his epilepsy was more or less under control. He was last seen when he was 5 years old and lived with his parents. He was frequently hospitalized because of complications related to his epilepsy. Ophthalmologic examination showed optic hypoplasia and nearly no visual reactions could be aroused. Cranial MRI showed significant hypoplasia of the pons and cerebellum (Figure 2).

**Patient 4** was born as the 1st child of a German couple in week 34 by Cesarean section following a pregnancy complicated by IUGR and preeclampsia/maternal edema with primary microcephaly (−2.57 SD) and relevant VSD. He suffered from respiratory and cardiac insufficiency (apnoe-bradycardy-syndrome), showed severe hypotonia from the beginning and was tube fed due to inability to swallow. He developed different types of seizures with burst suppression EEG from 2 months on. On examination at 6 months of age, microcephaly corresponded to −7.7 SD (corrected for prematurity), his skull was asymmetric and had a metopic ridge. He showed disproportionate short stature with a broad thorax, rhizomelic shortening of limbs, and slender lower legs. He had flexion contractures of fingers 2 and 4 on the left, and 4 on the right side, a high palate, broad alveolar ridges, mildly protruding ears of normal length with overfolded helices (Figure 1), undescended testes, mild shawl scrotum and a hemangioma on his back. At the same age, he received surgery for inguinal hernia and a PEG. At 15 months, he was still severely hypotonic and showed little psychomotor development. His seizures responded partly to levetiracetam and vigabatrin. Serial MRI at the age of 2 and 10 months showed severe pontocerebellar hypoplasia affecting particularly the cerebellar hemispheres, progressive cerebral atrophy and progressive hypomyelination (additional effects of complicated premature birth cannot be excluded) (Figure 2).

**Patient 5** was one of dizygotic twins, born as the 1st child in week 36 after a pregnancy established by egg cell donation and ICSI, and complicated by maternal diabetes and polyhydramnios. Decelerated growth of head circumference in fetus 1 was mentioned prenatally beginning at 20 weeks of pregnancy. OFC at birth corresponded to the 3rd centile. Patient 5 showed progressive microcephaly up to −6.5 SD during the first half year of life. He developed epileptic spasms at the age of 3 months but his EEG did not show hypsarrhythmia. The treatment could reduce the frequency of seizures. His psychomotor development was moderately retarded at the age of 6 weeks. Hearing impairment was suspected but could not be validated. Myopia was diagnosed at 6 months. Examination at age 7 months revealed severe global developmental delay with severe muscular hypertonia and absence of social interaction, reflective smiling or visual fixation. Dysmorphic signs comprised a broad nasal bridge, epicanthal folds, a long philtrum, retromicrognathia, and fleshy uplifted ear lobules (Figure 1). MR images showed a small brain with significant pontocerebellar hypoplasia affecting the cerebellar vermis and hemispheres equally (Figure 2).

**Patient 6** was born at term following an uneventful pregnancy as the 2nd child of an Iranian mother and a German father with microcephaly (−2.9 SD)*.* His mother reported two previous miscarriages. He showed progressive microcephaly during his 1st year of life and global developmental delay from the beginning. At the age of 11 months, his motor development corresponded to 3 months and his Developmental Mental Index was < 50. He had an afebrile seizure but otherwise normal EEG, and his tonus was increased. He exhibited subtle facial dysmorphism (broad nasal bridge, epicanthal folds, long philtrum, prominent premaxilla, mild retrognathia, simple/thin auricle, normal ear length) (Figure 1) reminiscent of the facial phenotype described in *CASK*-mutation positive females [4]. MRI of the brain showed moderate pontocerebellar hypoplasia affecting the cerebellar hemispheres and vermis equally, but no further anomalies (Figure 2).

**Patient 7** was born with a normal OFC as the 2nd child of a nonconsanguineous German couple at 36 + 1 weeks of gestation. On examination at the age of 29 months, he showed microcephaly (−3.56 SD), short stature (−3.5 SD), global developmental delay, and a tonus regulation disorder. Facial dysmorphism included a prominent nasal bridge, thin upper lip, and a small pointed chin (Figure 1). He walked at the age of 30 months and learnt some words, however, he could not use them appropriately. At the age of 5;3 years, OFC and height corresponded to −2.5 SD approximately, he was able to run but fell frequently due to mild ataxia. He was restless, constantly moving and reported to be normotonic. He uttered sounds, no words and could not yet be trained in sign language. His development had not been tested formally. Brain MRI performed at 10 months showed mild hypoplasia of the pons, cerebellar vermis and hemispheres, and mildly simplified gyri (Figure 2).

**Patient 8** was born at term to a 40-year-old Belgian mother. OFC corresponded to −2 SD at birth. He showed failure to thrive, rapidly progressive secondary microcephaly up to −5 SD at age 20 months, and mild to moderate developmental delay without further neurologic signs. The only physical anomaly consisted of a flat smooth philtrum (Figure 1). Brain MRI at the age of 18 months showed mildly smaller frontal lobes and a small frontal part of the corpus callosum. Cerebellum and pons were normal (Figure 2). The mother of patient 8 had a history of special education for learning difficulties, her OFC being 53 cm (3rd centile). She had three girls and a boy from a previous marriage, two of the girls required special education; the boy and a full sister of patient 8 were healthy.

### Molecular karyotyping

DNA was isolated from leukocytes, lymphoblastoid, fibroblast and buccal cells by standard procedures. Copy number profiling for patient 7 was performed using the SurePrint G3 Custom CGH Microarray, 4x180K (G4125A, Agilent Technologies, Santa Clara, CA, USA), a high resolution 60-mer oligonucleotide based microarray with median overall probe spacing of about 13 kb. Labelling and hybridization of genomic DNA were performed according to the protocol provided by Agilent without enzymatic restriction. Data visualization and analysis was performed as described [13]. Copy number profiling for patient 8 was performed on a 60K oligonucleotide array (Agilent Technologies, Diegem, Belgium) according to the manufacturer’s instructions with minor modifications as described [14]. CNV evaluation was performed using the in-house developed software tool arrayCGHbase [15].

### Multiplex ligation-dependent probe amplification (MLPA)

SALSA MLPA P398 CASK probemix (version A1) (MRC-Holland, Amsterdam, The Netherlands) was used according to the manufacturer’s instructions to detect single and multiple exon deletions/duplications in the *CASK* gene (exons 1–23 and 25–27). MLPA results were analysed with the Sequence Pilot algorithm, version 4.0.1 (JSI Medical Systems, Kippenheim, Germany). A relative probe signal of 1 (100%) in a male sample DNA reflected one copy of target sequence of the *CASK*-specific probe. Mosaic deletions of a probe’s recognition sequence led to a significant reduction in relative peak area (by 35-50% or higher). Relative signals of 1.5 (150%) of *CASK*-specific probes indicated two copies of the respective target sequences.

### Sequencing of *CASK*

The coding region of the *CASK* gene (27 exons) [GenBank:NM_003688] was amplified from genomic DNA. Primer sequences are available on request. Amplicons were directly sequenced using the ABI BigDye Terminator Sequencing Kit (Applied Biosystems, Darmstadt, Germany) and an automated capillary sequencer (ABI 3500; Applied Biosystems). Sequence electropherograms were analysed using Sequence Pilot software (JSI Medical Systems).

### Fluorescence *in situ* hybridization (FISH)

Metaphase spreads from peripheral blood lymphocytes were prepared by standard procedure and counterstained using 4′,6-diamidino-2-phenylindole (DAPI). BAC (RP11 human BAC library) and fosmid clones (WIBR-2 human fosmid library [G248P8]) were received from the BACPAC Resource Center, Children’s Hospital Oakland, CA, USA. BAC and fosmid DNA preparation and labelling as well as fluorescence microscopy and analysis of images was performed as previously described [16].

### Cell culture

Fibroblast cells were cultured in Dulbecco’s modified Eagle medium (DMEM; Life Technologies, Darmstadt, Germany) and lymphoblastoid cell lines in Roswell Park Memorial Institute 1640 medium (RPMI 1640; Life Technologies), both supplemented with 10% (v/v) fetal bovine serum (FBS; PAA Laboratories, Cölbe, Germany) and penicillin-streptomycin (100 U/ml and 100 μg/ml, respectively; Life Technologies) and incubated at 37°C in a humidified atmosphere with 5% CO_2_.

### Transcript analysis and cloning

Total RNA of patient 5 and three healthy males was extracted by using the PAXgene Blood RNA Kit (PreAnalytiX/QIAGEN, Hilden, Germany) according to the manufacturer’s instructions. Total RNA was extracted from cultured primary fibroblasts of patients 1, 2 and 5 and three healthy individuals as well as from lymphoblastoid cells of patient 8 and one healthy individual (RNeasy Mini Kit, QIAGEN). 1 μg of RNA was reverse transcribed into cDNA (Superscript II®; Invitrogen, Karlsruhe, Germany) using random hexanucleotides (Invitrogen) according to the manufacturer’s protocol. RT-PCR fragments were obtained by using different primer combinations according to standard PCR protocols (see online Additional file 1: Table S1). PCR products were cloned into pCR2.1 TOPO TA Cloning® Vector (Invitrogen). *E.coli* clones were subjected to colony PCR and PCR products from individual clones were sequenced.

### Antibodies and reagents

The following primary antibodies and dilutions were used: rabbit anti-CASK antibody (named anti-CASK2 antibody; WB 1:500; Cell Signaling Technology, Danvers, MA, USA), mouse anti-CASK antibody (named anti-CASK1 antibody; WB 1:1000; Millipore, Schwalbach, Germany), mouse anti-α-tubulin antibody (clone DM 1A; WB 1:7500; Sigma-Aldrich, Taufkirchen, Germany). As secondary antibodies, horseradish peroxidase-conjugated anti-mouse and anti-rabbit antibodies (1:1000–1:10000 dilution; GE Healthcare, Munich, Germany) were used.

### Immunoblotting

Cells were washed with ice-cold 1x PBS and harvested in modified RIPA buffer (150 mM NaCl, 50 mM Tris–HCl, pH 8.0, 0.5% sodium desoxycholate [w/v], 1% Nonidet P40 [v/v], 0.1% sodium dodecyl sulfate [w/v]) containing protease inhibitor cocktail (Roche, Mannheim, Germany). Cell debris was removed by centrifugation at 14000 rpm for 10 minutes at 4°C and protein solutions were supplemented with sample buffer. Proteins were separated on SDS-polyacrylamide gels and transferred to PVDF membranes. Following blocking (20 mM Tris–HCl, pH 7.4; 150 mM NaCl; 0.1% Tween-20; 4% non-fat dry milk) and washing (20 mM Tris–HCl, pH 7.4; 150 mM NaCl; 0.1% Tween-20), membranes were incubated in primary antibody solution (20 mM Tris–HCl, pH 7.4; 150 mM NaCl; 0.1% Tween-20; 5% BSA or 0.5% non-fat dry milk) containing the appropriate antibody. Next, membranes were washed and incubated with the peroxidase-coupled secondary antibody. After final washing, immunoreactive proteins were visualized using the Immobilon Western Chemiluminescent HRP Substrate (Millipore).

## Results

### *CASK* mutations

**Figure 3:**
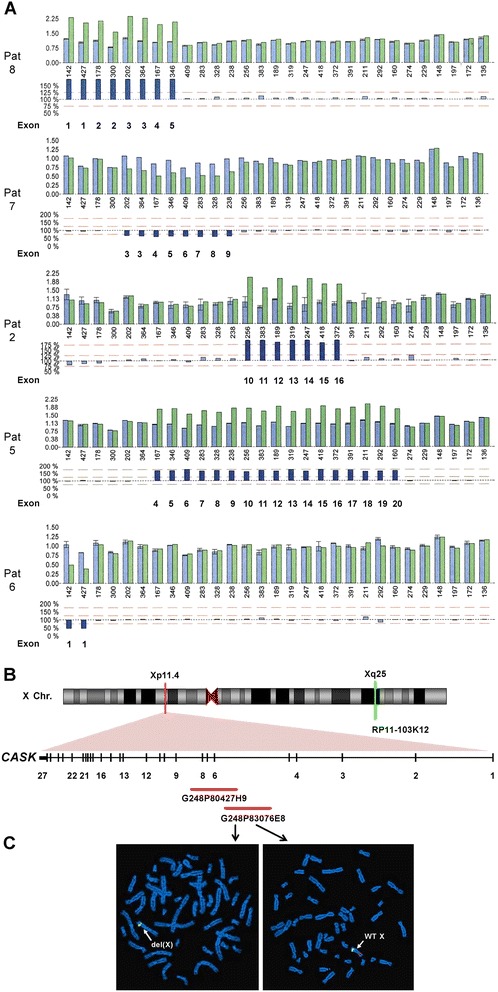
Partial *CASK* deletions and duplications. **A**. MLPA results on DNA isolated from leukocytes of patients 2, 5, 6, and 7 and on lymphoblastoid cell-derived DNA of patient 8. Bars in upper and lower histograms represent MLPA probes. Peak area histogram (upper histogram): blue bars represent the mean probe signals with standard deviations for three reference DNAs, except for patient 7 (one reference DNA); green bars: probe signals for patient DNA. Numbers below the bars indicate the amplicon size (bp) of each MLPA probe. Lower histogram: the bar for each probe represents the probe signal for patient DNA as percentage of the mean signal for the reference DNAs. Light blue bars represent percentages ranging from 75 to 125% (red dotted lines); deviations lower and higher than 75 and 125% are represented by dark blue bars. Deleted/duplicated *CASK* exons are indicated below the dark blue bars. **B**. Ideogram of the X chromosome is shown on the top. The Xp11.4 and the Xq25 regions are indicated by a red and a green bar, respectively. The *CASK* exon-intron structure is enlarged below: vertical lines represent exons and horizontal lines introns; selected exons are numbered. The two fosmid clones used to confirm somatic mosaicism of the *CASK* exon 3–9 deletion in patient 7 are indicated by red bars and names are given. BAC RP11-103K12 (Xq25) (indicated below the ideogram) was used as control probe. **C**. FISH with fosmid G248P83076E8 on a metaphase spread of patient 7 revealed a signal (red) on the wild-type (WT) X chromosome (arrow pointing to the normal X in the right picture), while the same probe did not hybridize on the X chromosome in another metaphase (arrow pointing to the del(X) in the left picture; see online Additional file 1: Table S1). RP11-103K12 gave a green signal in both metaphases.

High resolution molecular karyotyping was performed on a clinical basis on patients 7 and 8 and revealed copy number changes in Xp11.4: a possible mosaic deletion of ~160 kb (chrX: 41,496,539-41,655,017; hg19) in individual 7 (Additional file 1: Figure S1) and a duplication of 450–600 kb (chrX: 41,531,408-41,987,619; hg18) in patient 8 (Additional file 1: Figure S2). Both alterations cover part of the *CASK* gene, however, the duplication also encompassed a gene desert region of ~300 kb upstream of *CASK* (Additional file 1: Figure S2). We further characterized the two alterations by multiplex ligation-dependent probe amplification (MLPA). We confirmed the duplication of *CASK* exons 1–5 in DNA isolated from lymphoblastoid cells of patient 8 (Figure 3A) and identified his mother as carrier of the partial *CASK* duplication (data not shown). All three half sisters of individual 8 (including two half sisters with learning disabilities) did not carry the duplication as shown by molecular karyotyping; the half brother and full sister could not be tested. In patient 7, the mosaic deletion covering *CASK* exons 3–9 in DNA isolated from leukocytes and buccal cells was confirmed as the dosage of the seven exons was reduced by 50-60% compared to the other exons (Figure 3A and data not shown). FISH with the two fosmid clones G248P80427H9 and G248P83076E8 (Xp11.4) and BAC RP11-103K12 (Xq25) as control probe on metaphase spreads of patient 7 revealed that the *CASK* microdeletion was present in ~34% (22/65) of his leukocytes (Figures 3B and C and Additional file 1: Figure S2).

Sequence analysis of the 27 coding exons of *CASK* was performed on six male patients and revealed three pathogenic mutations: the nonsense mutation c.79C > T (p.R27*) in patient 4, the 5-bp deletion c.704_708delATAAG in patient 1 and a transition affecting the start codon, c.1A > G, in patient 3 (Table 1). The A-to-G change of the first ATG codon has previously been reported in a male with Ohtahara syndrome and cerebellar hypoplasia [9]. These three hemizygous *CASK* alterations were not present in the mothers of the patients and therefore occurred *de novo*.

MLPA for the *CASK* gene on the remaining three patients identified two intragenic duplications, one of exons 10–16 in patient 2 (Figure 3A) and one of exons 4–20 in patient 5 (Figure 3A). In patient 6, the relative peak area of the two probes for exon 1 was reduced by 50-60% indicating a mosaic deletion of this *CASK* exon (Figure 3A). Somatic mosaicism of the exon 1 deletion was confirmed in buccal cell-derived DNA of patient 6 by MLPA as the dosage of this exon was reduced by ~60% (data not shown). The two duplications were also detected in DNA isolated from the patients’ fibroblasts as well as in a third tissue (buccal cells) of individual 5 (data not shown). The mother of patient 2 did not carry the exon 10–16 duplication (data not shown), while the biological mother of patient 5 was not available.

### *CASK* transcript analysis

**Figure 4:**
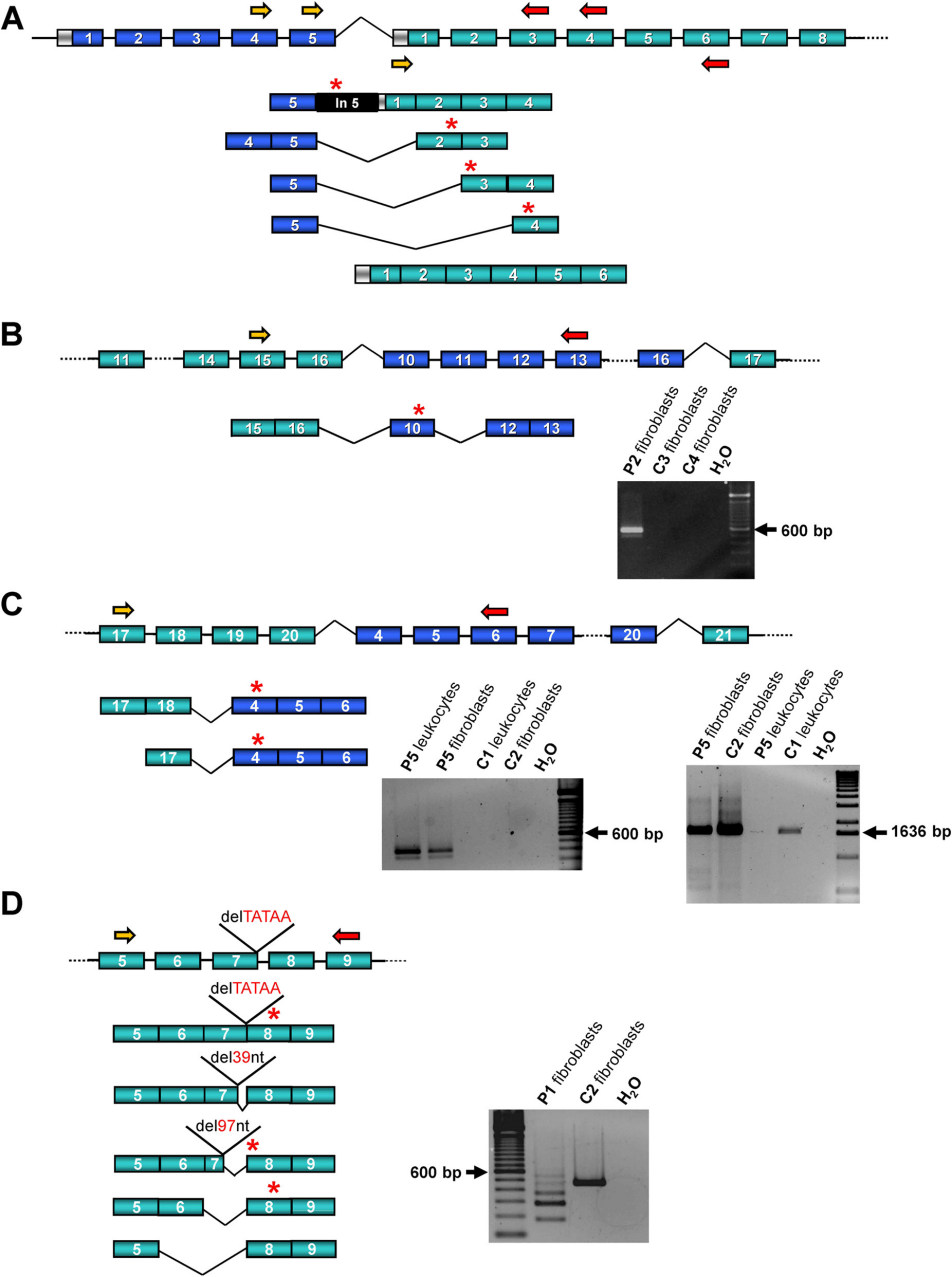
Transcript analysis of the *CASK* gene. **A**, **B**, **C** and **D**. Schematic representation of *CASK* transcript variants and representative RT-PCR products in patients 1, 2, 5 and 8. *CASK* exons are indicated by boxes: green boxes represent the coding region, blue boxes duplicated coding exons and the light grey box the 5′ untranslated region in exon 1. Primers used for RT-PCR experiments are represented by yellow (forward primer) and red (reverse primer) arrows (see online Additional file 1: Table S1). Premature termination codons are indicated by red stars above the respective transcript variant. *CASK* transcript analysis was performed using (**A**) lymphoblastoid cell-derived RNA of patient 8, (**B**) fibroblast-derived RNA of patient 2 (P2) and three healthy individuals (C2-C4), (**C**) leukocyte- and fibroblast-derived RNA of patient 5 (P5) and two healthy individuals (C1, C2) and (**D**) fibroblast-derived RNA of patient 1 (P1) and one healthy individual (C2). RT-PCR products are shown on the right for patients 1, 2 and 5 and healthy individuals as controls. **C**. A *CASK* fusion transcript was amplified from RNA isolated from both leukocytes and fibroblasts of patient 5 (left representative agarose gel electrophoresis picture), while a band corresponding to *CASK* wild-type transcript (from exon 3 to 21) was only generated from fibroblast-derived RNA of the patient (right representative agarose gel electrophoresis picture). A water control (H_2_O) was used in each RT-PCR reaction. The bright 600 bp reference band of the 100 bp DNA ladder and the 1636 bp band of the 1 kb DNA ladder are indicated by an arrow. del, deletion; nt, nucleotides; bp, base pairs.

To analyse if the duplicated *CASK* exons (1–5, 4–20 and 10–16) in patients 8, 5 and 2 were arranged in tandem and disrupt the integrity of the gene, we performed RT-PCR analysis to characterize *CASK* transcripts in the three patients. In patient 8 with the exon 1–5 duplication, we identified four different aberrant mRNAs in which exon 5 was directly spliced to exon 2, exon 3 or exon 4, and the fourth transcript contained exon 5, part of intron 5, and exons 1–4 indicating an in tandem duplication (Figure 4A). All these *CASK* transcripts harboured a premature termination codon (Figure 4A). We also amplified a *CASK* mRNA variant consisting of exons 1–6 and harbouring the 5′ untranslated region and start codon (Figure 4A). This mRNA could lead to the production of normal CASK protein.

In individuals 2 and 5, we amplified specific mRNA junction fragments that were not obtained in healthy individuals indicating an intragenic in tandem duplication (Figure 4B and C). Sequencing of the aberrant *CASK* transcript in patient 2, who carried the exon 10–16 duplication, revealed that exon 16 was spliced to exon 10, followed by exons 12 and 13. This *out-of-frame CASK* transcript was found in both RNA isolated from leukocytes and fibroblasts and contained a premature termination codon in exon 10 (Figure 4B). Cloning and sequencing of the junction fragments obtained from leukocyte- and fibroblast-derived RNA of patient 5 detected aberrant splicing of exon 17 or exon 18 to exons 4 and 5 indicating that exons 19 and 20 were skipped in the majority of transcripts, and exons 18–20 in some of them (Figure 4C). Both *CASK* transcript variants (exon 17-4-5 and exon 18-4-5) contained a premature stop codon in exon 4 (Figure 4C).

The 5-bp deletion c.704_708delATAAG in patient 1 leads to a frameshift and introduction of a premature termination codon (p.(K236Efs*10)). However, this deletion affects the last five nucleotides of exon 7 suggesting that recognition of the adjacent splice donor site in the pre-mRNA could be disturbed by the spliceosome. We tested this hypothesis by performing RT-PCR on RNA isolated from fibroblasts of patient 1 that yielded multiple amplicons of different sizes compared with a single PCR product generated from control RNA (Figure 4D). Amplicon cloning followed by sequencing of a total of 53 clones revealed five different *CASK* transcript variants in fibroblasts of patient 1: in the major transcript form exon 7 was skipped; minor forms included transcripts containing exons 5 to 8 with the 5-bp deletion in exon 7, transcripts lacking both exons 7 and 8 as well as transcripts with a shortened exon 7 (skipping of 39 bp or of 97 bp at the 3′ end) (Figure 4D and Additional file 1: Figure S3). Three out of the five *CASK* mRNA variants harboured a premature termination codon in exon 8 (Figure 4D).

### CASK protein analysis

**Figure 5:**
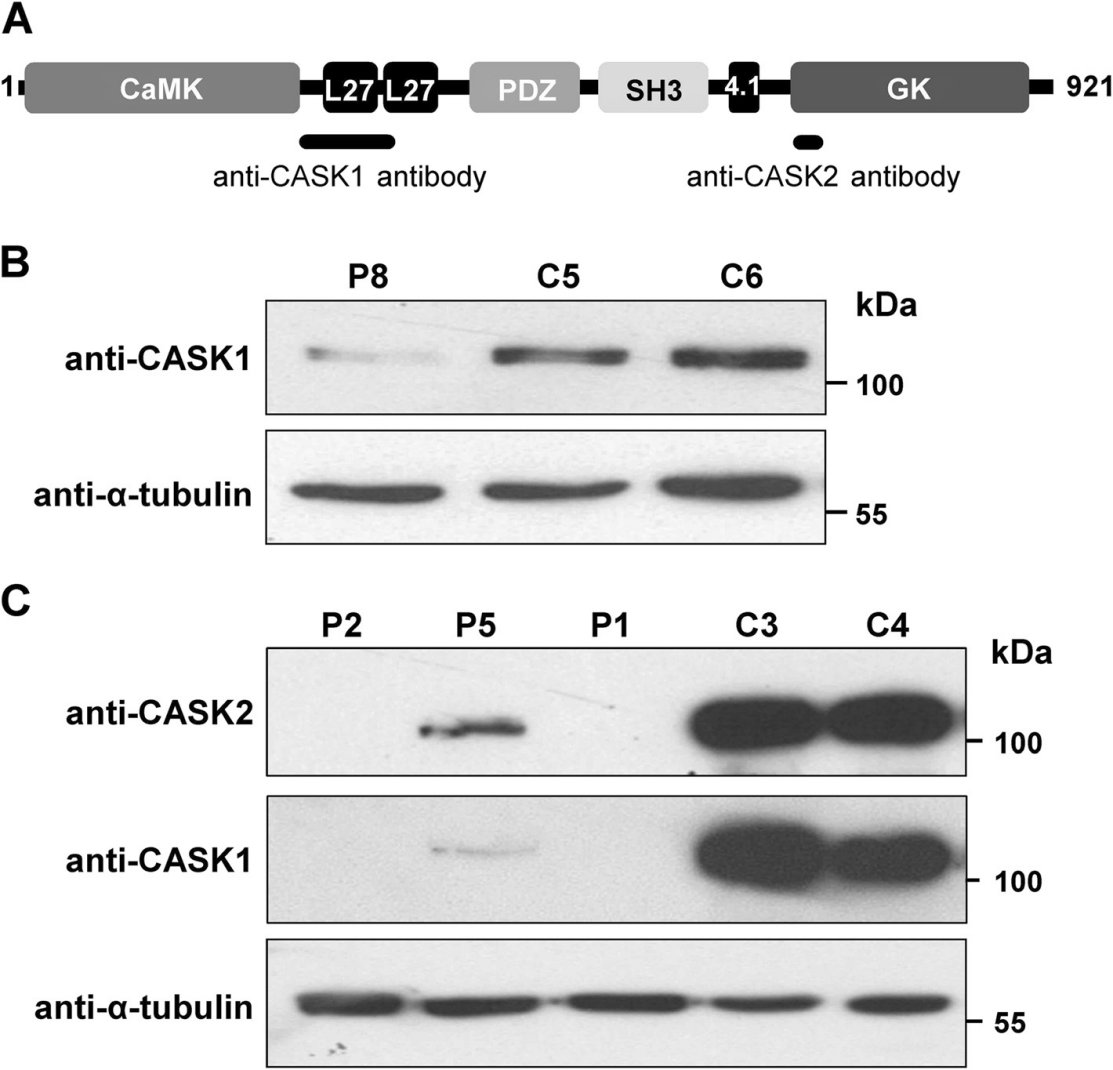
Expression of CASK protein. **A**. Domain structure of the longest CASK isoform [GenBank:NP_003679.2]. Domains are represented by boxes in black and different shades of grey. The number on the left and right indicates the first and last amino acid residue of this CASK isoform, respectively. Black bars below the domain structure show the two regions used as immunogen to produce anti-CASK1 and anti-CASK2 antibodies. CaMK: calmodulin-dependent kinase-like domain; L27: LIN-2 and LIN-7 interaction; PDZ: PSD-95-Dlg-ZO1; SH3: Src homologous 3; 4.1: protein 4.1 interaction; GK: guanylate kinase. **B**. Total cell lysates of lymphoblastoid cells derived from patient 8 (P8) and two healthy male individuals (C5 and C6) were subjected to SDS-PAGE and immunoblotting. The amount of total CASK protein was monitored by using the anti-CASK1 antibody (Millipore) (upper panel). Anti-α-tubulin antibody was used to control for equal loading (lower panel). **C**. Total fibroblast cell lysates from patients 1, 2 and 5 (P1, P2 and P5) and two healthy male individuals (C3 and C4) were analysed by immunoblotting using the anti-CASK1 antibody (Millipore) (middle panel) and the anti-CASK2 antibody (Cell Signaling) (upper panel). Equal loading was controlled by an anti-α-tubulin antibody (lower panel).

To analyse if the male patients with a *CASK* mutation express normal and/or a truncated protein variant, we performed immunoblot analysis with protein lysates prepared from lymphoblastoid cells of patient 8 and fibroblasts of individuals 1, 2 and 5. No cell line was available for the other four patients. We initially used an anti-CASK antibody which detected the CASK-specific domain from amino acids 318–415 (named anti-CASK1 antibody; Figure 5A). In lymphoblastoid cells of patient 8, we observed a faint band with a molecular weight of ~110 kDa corresponding to wild-type protein (Figure 5B). However, the amount of CASK was drastically decreased in patient 8 compared with two healthy individuals (Figure 5B). In fibroblast cells of patient 2 with the *CASK* exon 10–16 duplication and of patient 1 with the splice mutation c.704_708delATAAG, no CASK protein could be detected, while a prominent band corresponding to CASK wildtype was visible in two fibroblast cell lines of healthy individuals (Figure 5C). In contrast, a faint band with the expected molecular weight of wild-type CASK (~110 kDa) was observed in the fibroblast lysate of patient 5 (Figure 5C). To confirm that this band was no artefact, we probed the same lysates of the three patients 1, 2 and 5 as well as of two healthy males with another anti-CASK antibody, recognizing residues surrounding threonine 774 (COOH terminus) (anti-CASK2 antibody; Figure 5A). Similar to the anti-CASK1 antibody, this antibody also identified a small amount of wild-type CASK protein in the fibroblast lysate of individual 5 (Figure 5C), suggesting that fibroblast cells of this patient still generate some wild-type *CASK* transcripts that are translated to produce normal protein. These data indicate that patient 5 is a somatic mosaic for the intragenic *CASK* exon 4–20 duplication, but with a high level mosaicism. We confirmed somatic mosaicism for this *CASK* alteration by amplification of a wild-type *CASK* transcript comprising the coding region of exons 3–21 (1700 bp) in fibroblast-derived RNA of patient 5, while no such amplicon could be generated from RNA isolated from his leukocytes (Figure 4C).

## Discussion

**Figure 6:**
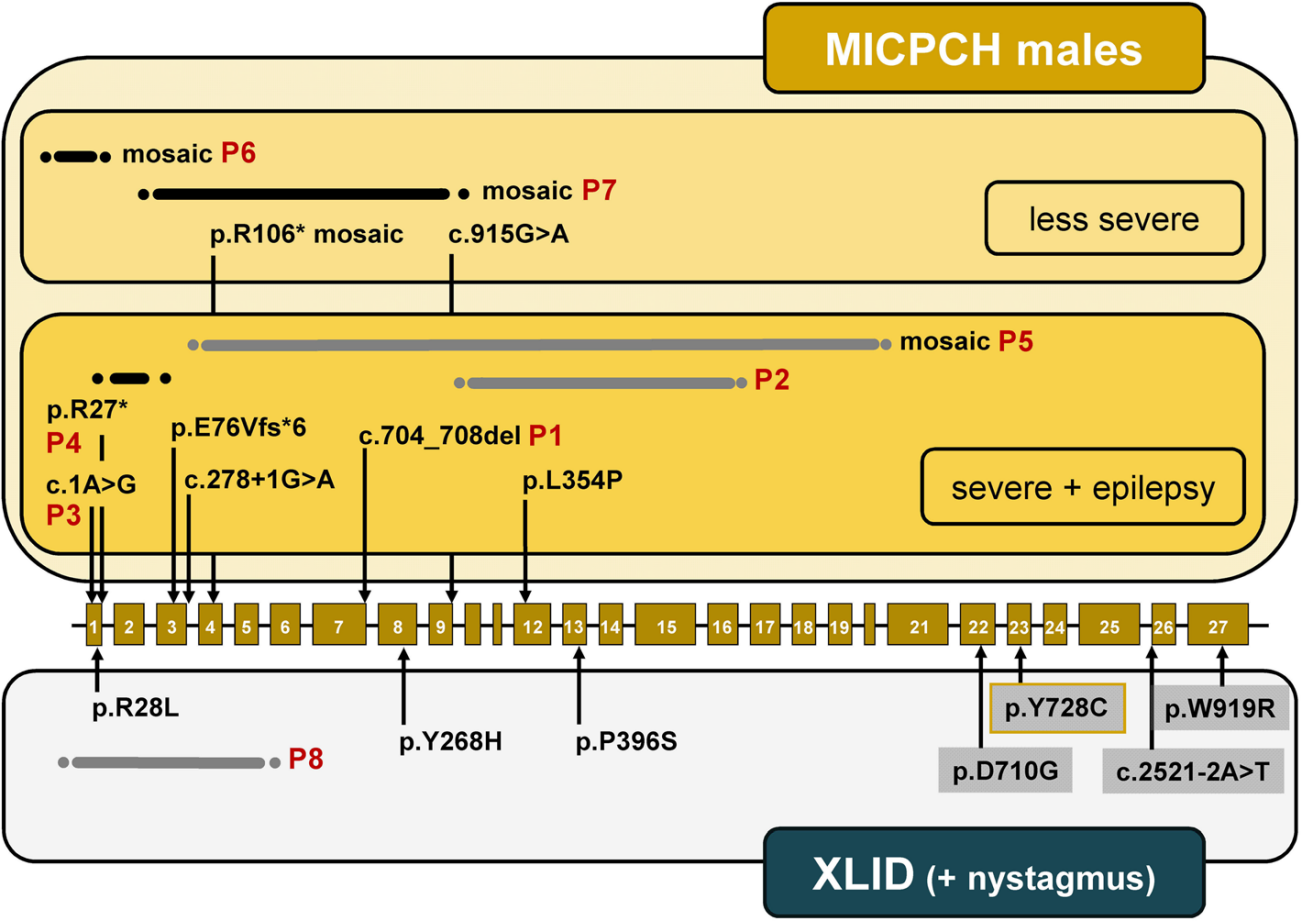
Summary of *CASK* mutations in males. Exons of *CASK* are represented as light brown boxes and introns as black bars. The exon-intron structure is not drawn to scale. *CASK* mutations are given at the nucleotide level (for variants affecting splicing and for variants for which the prediction on protein level is not possible) or protein level. The arrows point to the position of the mutation within the exon or intron. Duplications of *CASK* exons are represented by grey bars and deletions by black bars; dots indicate that the exact duplication/deletion breakpoints have not been determined. Mutations associated with microcephaly and pontocerebellar hypoplasia (MICPCH) with/without epilepsy are grouped above the exon-intron structure (yellow background); mutations in individuals with severe MICPCH with epilepsy and less severe MICPCH are differentiated by dark and light yellow background, respectively. Mutations in males with X-linked intellectual disability (XLID) are shown below the exon-intron structure (light grey background), those associated with nystagmus have a dark grey background. The yellow framed p.Y728C change was identified in two brothers, one with MICPCH and nystagmus and one with ID, microcephaly and nystagmus. P1-P8: Numbering of patients 1–8 as described in this study.

We present eight new, unrelated male patients with a *CASK* alteration and a detailed characterization of their clinical features, genetic and molecular data. Based on our findings and previously published results on sporadic and familial cases (Table 1), we propose the existence of three phenotypic groups in males (Figure 6) which can be clinically distinguished but represent a clinical continuum from the severe to the mild end of the spectrum.

(i) sporadic male patients with MICPCH and severe epileptic encephalopathy:
  This group includes ten individuals so far (patients 1–5 reported here and five patients reported previously, Table 1). *CASK* alterations in these patients likely represent germline mutations, except for patient 5, and include a missense mutation, a nonsense mutation, a splice site mutation, a mutation affecting the start codon in two individuals, a 2 bp and a 5 bp deletion, two intragenic duplications of multiple exons and a deletion of exon 2 (this report and refs. [2,5,8,9]). Transcript analysis in our patients 1, 2 and 5 and the patient with the exon 2 deletion revealed that the mutations severely disturb the integrity of the *CASK* gene as aberrant mRNAs with a premature termination codon were produced (this report and ref. [9]). In addition, wild-type CASK protein was absent in cells of patients 1 and 2 (this report) and the two patients with c.1A > G and the exon 2 deletion [9], further indicating that these males carry a *CASK* null allele. Patient 5 also has an inactivating *CASK* mutation, however, by immunoblotting we demonstrated a small amount of CASK protein with a molecular mass corresponding to that of wildtype in his fibroblasts. To confirm expression of wild-type CASK, we tried to amplify a *CASK* transcript harboring exons 3–21 from RNA isolated from leukocytes and fibroblasts of patient 5 and indeed generated an RT-PCR product from his fibroblast-derived RNA. This finding indicates that patient 5 has a high level somatic mosaicism of the exon 4–20 duplication. In accordance with this interpretation, no exon 3-to-21 transcript was amplified from leukocyte-derived RNA of patient 5 suggesting that expression of *CASK* is generally low in blood and/or the degree of somatic mosaicism of the exon 4–20 duplication is higher in leukocytes than in fibroblasts.
  In this group, the affected males have the most severe clinical presentation. Developmental delay is severe to profound; the eldest reported patients are 5 years of age and hardly show any development (patient 3 in Table 1 and patient 16 in ref. [5]). Similar to females with MICPCH, microcephaly is congenital or evolves rapidly during the first months of life and later becomes significant (up to −9 SD). MRI consistently shows significant or severe pontocerebellar hypoplasia. Other brain malformations include rarefication of gyri, (progressive) cortical atrophy and progressive hypomyelination but no further supratentorial involvement. Seizures manifest as Ohtahara syndrome, West syndrome, early myoclonic encephalopathy or just as unspecified intractable seizures. We conclude that *CASK* loss-of-function alterations cause severe epileptic encephalopathy in males but do not specifically underlie e.g. Ohtahara syndrome. Affected individuals frequently need a percutaneous endoscopic gastrostomy (PEG) for feeding and/or tracheostomy because of apnea. Congenital heart defects, in particular septal defects, are present in 4/10 patients and contractures, long slender fingers or shortening of limbs in two. 8 of 10 show retro-/micrognathia and fleshy uplifted ear lobules are seen in five patients (Figure 1).
  Male individuals with a severe *CASK* mutation clinically manifest with unspecific early epileptic encephalopathy and congenital or early postnatal and rapidly progressing microcephaly. Thus, the brain imaging findings are the major diagnostic clue and should prompt *CASK* testing, however, the differential diagnosis of other pontocerebellar hypoplasia (PCH) forms can be challenging [17]. For example, the *CASK*-mutation positive patient reported by Nakamura et al. [8] had been previously described with a clinical diagnosis of PCH type 3 [18], and it cannot be excluded that additional individuals with PCH may have been misclassified and are without a molecular diagnosis up to date.
(ii) MICPCH and a severe developmental disorder but without severe epilepsy:
  The *CASK* alterations in this group of five individuals (patients 6–7 reported here and three patients reported previously, Table 1) include severe mutations in mosaic state (nonsense mutation, exon 1 deletion and exon 3–9 deletion) as well as a partly penetrant splice mutation (c.915G > A, affecting the last nucleotide of exon 9) and a missense mutation (c.2183A > G, p.Y728C), both in the hemizygous state (this report and refs. [1,2,10]). The three patients with somatic mosaicism of the *CASK* mutation produce wild-type protein in a significant percentage of their cells, most likely reducing clinical severity in these cases.
  The phenotype of the males in this group is comparable to MICPCH in females. DD/ID (if specified) is severe, microcephaly is variable and the degree of pontocerebellar hypoplasia on MRI does not necessarily correlate with the degree of DD. Seizures have not been described in the five individuals, however, patient 5 reported in Najm et al. [1] deceased shortly after birth. The interpretation of clinical data of this patient group is limited by the small sample size and restricted data on patient 5 [1]. One individual, patient II-4 of family V in Hackett et al. [10], presented with nystagmus besides severe ID and cerebellar hypoplasia. His brother (patient II-2) shows a similar but milder phenotype, with nystagmus and mild microcephaly but no imaging of the brain has been reported.
(iii) mild to severe ID with or without nystagmus:
  The 21 cases of this group (patient 8 reported here and 20 cases published previously) are all familial and carry splice mutations or missense variants affecting amino acid residues in different CASK protein domains (Figure 6) [10-12]. Patient 8 harbors a duplication of exon 1 to 5 which was inherited from his mother. We identified multiple aberrant *CASK* transcripts in lymphoblastoid cells of this individual, however, we also amplified a *CASK* mRNA which contained exons 1 to 6 in the correct order, including part of the 5' untranslated region. Consistently, we detected a small amount of wild-type CASK protein in the patient’s cells indicating that some *CASK* transcripts are efficiently translated.
  In this group, microcephaly is documented only in individual 8 and in patient II.2 of family V [10]. Pontocerebellar hypoplasia or other brain anomalies can only be excluded in a minority of patients because imaging of the brain has often not been performed or reported. Various seizure disorders have been reported for seven males from four families, in none of them being severe. Nystagmus was observed in 12 patients. Short stature, facial dysmorphism, behavioural problems, tremor and/or hypotonia may be associated. Intrafamilial variability seems to be small, in particular with regard to eye findings and the degree of ID. In the family with the segregating c.83G > T mutation, the affected males have been described as having FG syndrome (FGS) [11,19], which is a heterogeneous X-linked condition initially described by Opitz and Kaveggia (Opitz-Kaveggia syndrome, MIM 305450) [20]. FGS1 is caused by a recurrent mutation in *MED12* (MIM 300188) [21], and has a well-defined phenotype consisting of DD/ID, hypotonia, dolichomacrocephaly, characteristic facies including small ears and hypertelorism, and congenital anomalies of the corpus callosum, heart and/or skeleton. The phenotype of the other FGS types, however, is poorly defined. Thus, we propose the clinical diagnosis of the males with the *CASK* mutation c.83G > T should be syndromic ID rather than FGS.
  Restricted clinical data are available for 13 carrier females from seven families. No abnormalities are reported in seven of these females, deriving from three families. Four carriers showed mild to severe ID, one had absence seizures, two tremor, and three nystagmus. MRI findings are given for one carrier only (no anomalies) who presented with ID and nystagmus.

The three phenotypic groups, however, represent a clinical continuum and at least two males link the groups: patient 5 with a high level mosaicism for the duplication of exons 4 to 20 and a less severe form of epilepsy links group I to II; and patient II.4 from family V with the missense mutation c.2183A > G (p.Y728C) [10], who represents the interface to group (iii) as he and his brother who carried the same mutation both had nystagmus.

*CASK* encodes the calcium/calmodulin (CaM)-dependent serine protein kinase which belongs to the membrane-associated guanylate kinase (MAGUK) protein family, the most prominent family of multidomain scaffolding proteins [22]. CASK has been shown to control synapse formation and activity by (i) presynaptic organization and regulation of neurotransmitter release, (ii) maintaining the morphology of dendritic spines and trafficking of ion channels to the postsynaptic site, and (iii) regulating the transcription of genes involved in cortical development by entering the nucleus [23,24]. However, the exact pathomechanism underlying the clinical spectrum ranging from MICPCH with severe epilepsy to mild ID is still unclear. In general, hemizygous *CASK* variants in males of phenotype group (iii) have been suggested to represent hypomorphic alleles [10,11], altering one or more of the multiple CASK functions while preserving others. An impact on protein structure and/or function has been demonstrated for the pathogenic *CASK* alterations p.Y728C and p.W919R, while the nature of the deleterious impact on CASK function remains to be determined for p.R28L, p.Y268H and p.P396S [25,26]. The two splice mutations c.2129A > G and c.2521-2A > T likely lead to the production of CASK proteins lacking several amino acid residues [10,11]. These mutant proteins may misfold, mislocalize, loose their capacity to bind CASK interaction partners and/or have a changed function. For example, the constitutively active CaM-kinase domain in the N-terminus of CASK [27] could be affected by the amino acid alteration p.R28L.

The nature of the *CASK* mutations found in males of phenotypic groups (i) and (ii) is quite different from that of group (iii) and corresponds to the mutation types found in females [2-5]. Indeed, germline and mosaic alterations consistently found in group (i) likely are null alleles as has been shown by *CASK* transcript analysis and immunoblotting demonstrating the absence of CASK protein in different cell types of severely affected individuals (Figures 4 and 5; ref. [9]). The non-synonymous *CASK* mutation c.1061T > C/p.L354P found in a 5-year-old male patient of this group is possibly a loss-of-function mutation in view of his severe phenotype, but the functional consequences of this alteration have not been investigated [5]. The small amount of normal CASK protein in patient 5 correlated well with the presence of *CASK* wild-type transcript in his fibroblasts (Figure 4C). Somatic mosaicism for a severe *CASK* mutation attenuates the phenotype in males, as shown for patients 6 and 7 in our cohort and patient 12 described by Burglen et al. [2]. However, the level of somatic mosaicism can be high, as in patient 5, and may mimic a germline mutation. Patients who are somatic mosaics of a *CASK* inactivating mutation are expected to produce wild-type CASK protein in a small percentage of their cells, as demonstrated for patient 5. Obviously, this scenario did not protect them from developing pontocerebellar hypoplasia that can readily be detected on brain imaging. Interestingly, in lymphoblastoid cells of patient 8 who carried the *CASK* exon 1–5 duplication, a small amount of wild-type CASK protein was observed. However, in contrast to somatic mosaics, this patient still produces some wild-type CASK in all of his cells and not only in a portion of cells. It can be hypothesized that the small amount of functional CASK was sufficient for an apparently normal structural development of the brain in patient 8, but led to microcephaly and ID, similar to the missense and splicing mutations described in males with X-linked non-syndromic/syndromic ID.

## Conclusions

In summary, we describe three phenotypic groups forming an overlapping spectrum of *CASK*-related disorders in males: group (i) includes the most severely affected males, group (ii) is a mitigation of group (i), and group (iii) includes males with variable ID and facultative clinical features. Our detailed analysis of the impact of mutations on *CASK* pre-mRNA splicing and protein expression indicates a genotype-phenotype correlation: inactivating *CASK* germline mutations are associated with the most severe phenotype (MICPCH with severe epileptic encephalopathy), while in the mosaic state these mutations result in an attenuated phenotype (MICPCH) that can also be caused by partly penetrant *CASK* mutations. *CASK* alterations leaving the protein intact, but with a reduced functionality or a reduction in protein level, are found in males with highly variable X-linked ID. We recommend *CASK* testing in male patients with the combination of DD/ID or epileptic encephalopathy, postnatal microcephaly (< −3 SD) and pontocerebellar hypoplasia. Sequencing of exons 22–27 may also be considered in patients with ID and nystagmus.

## Additional file

Additional file 1: Figure S1. Detection of a 150 kb *de novo* deletion at Xp11.4 targeting *CASK* in patient 7 by array CGH. Below the ideogram of the X chromosome, genomic copy numbers are shown along the X chromosome (Agilent 180K array). In the lower part the genomic profiles from individual 7 (upper line), his mother (middle line) and father (lower line) at Xp11.4 is zoomed in showing the deletion within the *CASK* gene in the patient (yellow shaded box, from 41,496,539 to 41,655,017 according to hg19). This deletion is not present in the parents. **Figure S2.** Detection of a duplication of 450-600 kb (chrX: 41,531,408-41,987,619; hg18) in patient 8 (Agilent 60K array). Duplicated probes are indicated in green on their genomic position on the X chromosome. Probes with a normal copy number are indicated in black. The lines represent the segmentation algorithm that was used (CBS). Below the enlarged ideogram, the genes in the duplicated interval and in the surrounding region are indicated in blue. **Table S1.** Primer sequences and primer combinations for *CASK* transcript analysis in patients 1, 2, 5 and 8. **Table S2.** FISH with two fosmid clones covering the mosaic *CASK* exon 3-9 deletion in patient 7. **Table S3.** Quantification of different *CASK* transcript variants in patient 1.
