# Supplementary material for: Phenotypic and molecular insights into CASK-related disorders in males
Source: Orphanet J Rare Dis. 2015 Apr 12;10:44. doi: 10.1186/s13023-015-0256-3 (PMC4449965; doi:10.1186/s13023-015-0256-3)
Supplement: Additional file 1: Figure S1. — Detection of a 150 kb de novo deletion at Xp11.4 targeting CASK in patient 7 by array CGH. Below the ideogram of the X chromosome, genomic copy numbers are shown along the X chromosome (Agilent 180K array). In the lower part the genomic profiles from individual 7 (upper line), his mother (middle line) and father (lower line) at Xp11.4 is zoomed in showing the deletion within the CASK gene in the patient (yellow shaded box, from 41,496,539 to 41,655,017 according to hg19). This deletion is not present in the parents. Figure S2. Detection of a duplication of 450-600 kb (chrX: 41,531,408-41,987,619; hg18) in patient 8 (Agilent 60K array). Duplicated probes are indicated in green on their genomic position on the X chromosome. Probes with a normal copy number are indicated in black. The lines represent the segmentation algorithm that was used (CBS). Below the enlarged ideogram, the genes in the duplicated interval and in the surrounding region are indicated in blue. Table S1. Primer sequences and primer combinations for CASK transcript analysis in patients 1, 2, 5 and 8. Table S2. FISH with two fosmid clones covering the mosaic CASK exon 3-9 deletion in patient 7. Table S3. Quantification of different CASK transcript variants in patient 1. [file 13023_2015_256_MOESM1_ESM.doc]

# Additional File


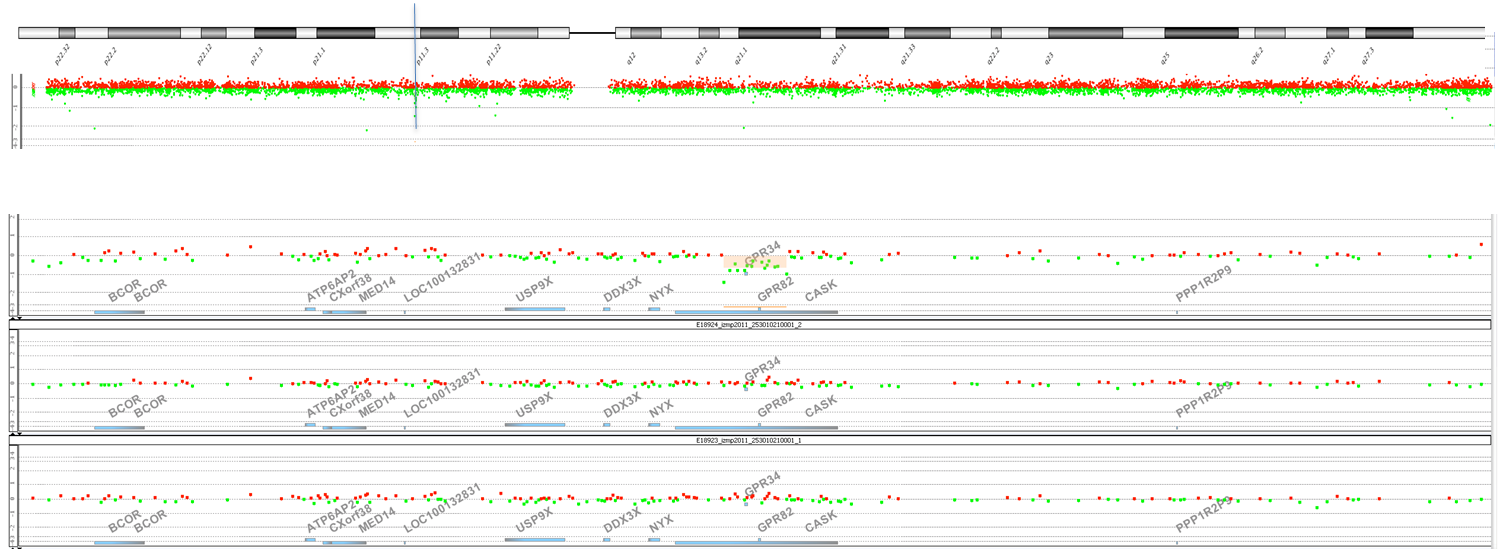


**Figure S1:** Detection of a 150 kb *de novo* deletion at Xp11.4 targeting *CASK* in patient 7 by array CGH

Below the ideogramm of the X chromosome, genomic copy numbers are shown along the X chromosome (Agilent 180 k). In the lower part the genomic profiles from individual 7 (upper line), his mother (middle line) and father (lower line) at Xp11.4 is zoomed in showing the deletion within the *CASK* gene in the patient (yellow shaded box, from 41,496,539 to 41,655,017 according to hg19). This deletion is not present in the parents.


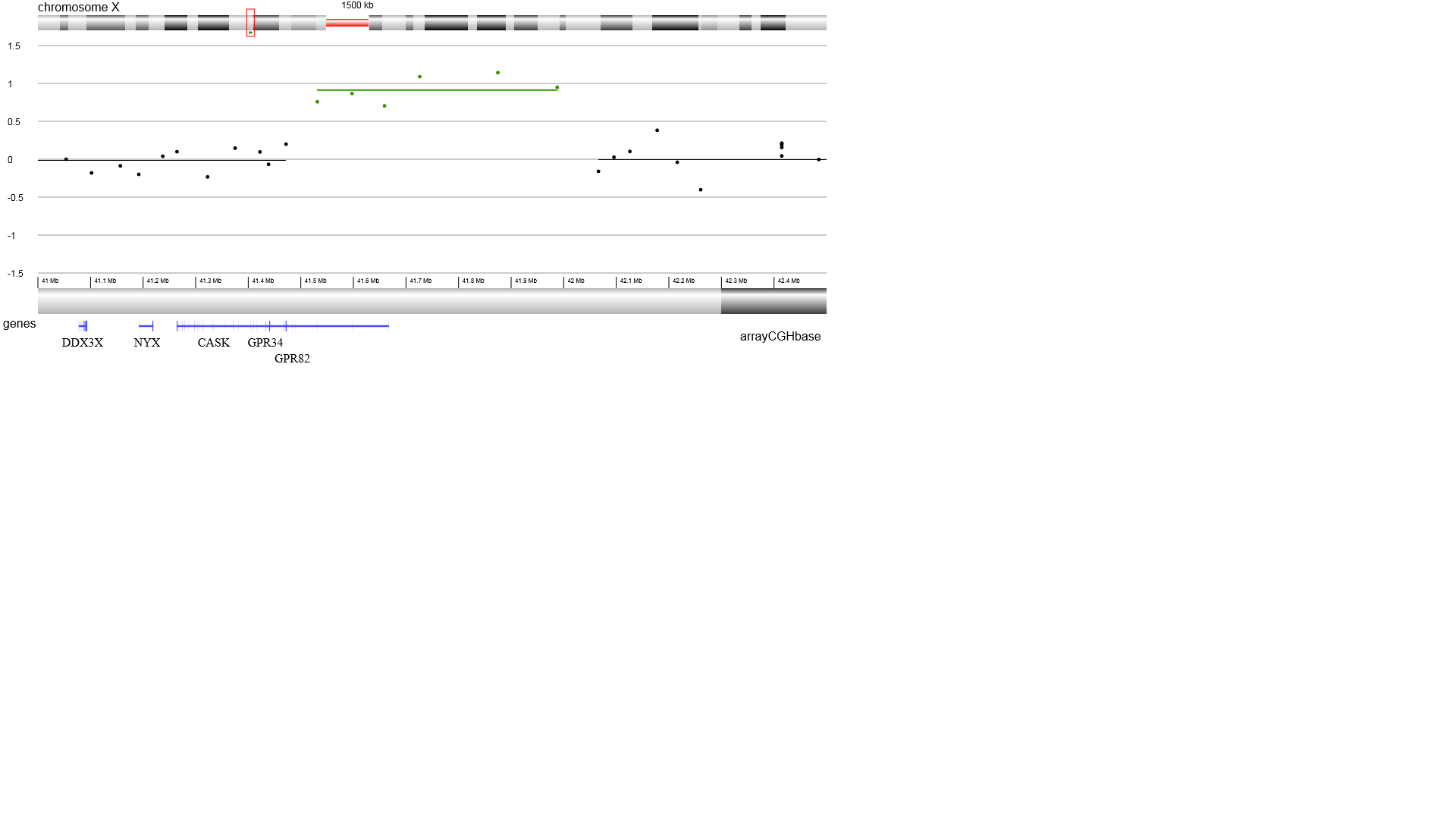


**Figure S2:** Detection of a duplication of 450-600 kb (chrX: 41,531,408-41,987,619; hg18) in patient 8 (Agilent 60K array)

Duplicated probes are indicated in green on their genomic position on the X chromosome. Probes with a normal copy number are indicated in black. The lines represent the segmentation algorithm that was used (CBS). Below the enlarged ideogram, the genes in the duplicated interval and in the surrounding region are indicated in blue.

# Table S1: Primer sequences and primer combinations for *CASK* transcript analysis in patients 1, 2, 5 and 8

| **exon** | **forward primer** | **5’ 🡪 3’ sequence** | **exon** | **reverse primer** | **5’ 🡪 3’ sequence** | **patient** |
| --- | --- | --- | --- | --- | --- | --- |
| 1  (5’UTR) | cCASK5UTR_F | GTCCTGAGGGGAGGAGGCGAT | 6 | cCASKex6_R | TCCTCCAGCTACAAGTCCAGACTC | 8 |
| 3 | cCASKex3_F | GGAAGCCAGTATCTGTCATATGC | 21 | CASK_21Rint | CCTGCCACCAATTATGATCATCC | 5 |
| 4 | cCASKex4_F | TTGAAATCGTAAAGCGAGCTGACG | 3 | cCASKex3_R | AGCATTCCATCTGAGCTATATGTCTC | 8 |
| 5 | cCASKex5_F | GATACTGGAAGCTCTACGCTAC | 4 | cCASKex4_R | CTGGCTACAGCTTCACTGTACAC | 8 |
| 5 | CASK_5_F | AGACAGATACTGGAAGCTCTAC | 9 | CASK_9_R | TTTCCTCCTTGCATTGAATTTCC | 1 |
| 15 | cCASK_15F | ACTTCAGACTCACGACGTAGTG | 13 | cCASK_13R | CAATACCTCTTTGGCTCTCTGTACTG | 2 |
| 17 | CASK_17_F | CACTTCATGTTGGTGATGAAATTC | 6 | CASK_6_R | CTCCAGCTACAAGTCCAGAC | 5 |

5’UTR: 5’ untranslated region

# Table S2: FISH with two fosmid clones covering the mosaic *CASK* exon 3-9 deletion in patient 7

| **Fosmid** | ***CASK* exons/introns** | **Chromosomal region** | **Total number of analysed metaphases** | **Signal on X chromosome (number of metaphases)** | **No signal on X chromosome (number of metaphases)** |
| --- | --- | --- | --- | --- | --- |
| G248P80427H9 | exons 6-8 | Xp11.4 | 35 | 22 | 13 |
| G248P83076E8 | intron 5 | Xp11.4 | 30 | 21 | 9 |

BAC RP11-103K12 was used as a control probe to label the X chromosome in all metaphases.

# Table S3: Quantification of different *CASK* transcripts in patient 1

| ***CASK* transcript variants** | **Number of transcripts after cloning and sequencing** |
| --- | --- |
| Exons 5 – 8 with c.704_708del in exon 7 | 6 |
| Exons 5, 6, 7 Δ39nt at 3' end, 8 | 5 |
| Exons 5, 6, 7 Δ97nt at 3' end, 8 | 3 |
| Exons 5, 6, 8 | 31 |
| Exons 5, 8 | 8 |
| Total number | 53 |

Δ: deletion; nt: nucleotides
